# Tropicalization strengthens consumer pressure on habitat-forming seaweeds

**DOI:** 10.1038/s41598-017-00991-2

**Published:** 2017-04-11

**Authors:** Salvador Zarco-Perello, Thomas Wernberg, Tim J. Langlois, Mathew A. Vanderklift

**Affiliations:** 1 School of Biological Sciences and UWA Oceans Institute, The University of Western Australia, Crawley (Perth), 6009 Western Australia Australia; 2 Commonwealth Scientific and Industrial Research Organisation (CSIRO), Oceans and Atmosphere Flagship, Indian Ocean Marine Research Centre, Crawley, Western Australia 6009 Australia

## Abstract

Ocean warming is driving species poleward, causing a ‘tropicalization’ of temperate ecosystems around the world. Increasing abundances of tropical herbivores on temperate reefs could accelerate declines in habitat-forming seaweeds with devastating consequences for these important marine ecosystems. Here we document an expansion of rabbitfish (*Siganus fuscescens*), a tropical herbivore, on temperate reefs in Western Australia following a marine heatwave and demonstrate their impact on local kelp forests (*Ecklonia radiata*). Before the heatwave there were no rabbitfish and low rates of kelp herbivory but after the heatwave rabbitfish were common at most reefs and consumption of kelp was high. Herbivory increased 30-fold and kelp abundance decreased by 70% at reefs where rabbitfish had established. In contrast, where rabbitfish were absent, kelp abundance and herbivory did not change. Video-analysis confirmed that rabbitfish were the main consumers of kelp, followed by silver drummers (*Kyphosus sydneyanus*), a temperate herbivore. These results represent a likely indirect effect of the heatwave beyond its acute impacts, and they provide evidence that range-shifting tropical herbivores can contribute to declines in habitat-forming seaweeds within a few years of their establishment.

## Introduction

A general poleward shift in distribution of marine species due to ocean warming was forecast decades ago^1–4^. Since then, numerous studies have documented unusual and increasing occurrences of warm-water species of diverse phyla at higher latitudes^5–8^, such as hermatypic corals^9^, echinoderms^10, 11^, mollusks^7^ and fish^11–14^. Range expansions have been accentuated in regions where intensifying warm currents from the tropics accelerate warming at temperate latitudes^11, 15^. Such currents have increased the arrival of warm-water vagrant species and allowed them to remain at higher latitudes for longer periods of time, attain higher abundances and sometimes establish permanent populations^11, 16^. As a consequence, the proportion of tropical species increase, resulting in a ‘tropicalization’ of the community. In some ecosystems this change in species composition has led to increased competition and species displacement^17, 18^, modification of food-webs and ecosystem functioning^19^, and regime shifts caused by the arrival of habitat-modifying species, such as herbivorous urchins or fish^8, 20^.

Increasing abundances of tropical herbivorous fish on temperate reefs will prompt major changes and threaten the services that these ecosystems provide^11^. Temperate reefs are characterised by high abundances of habitat-forming seaweed, typically dominated by kelp^21^, which support high levels of biodiversity and multiple fishery resources^22^. Rates of herbivory by fish are generally low in temperate reef systems^23–26^, although in some places a few fish species have shown high-intensity but small-scale effects on seaweed communities^27–30^. In contrast, herbivorous fish play a critical role in healthy tropical reef ecosystems where they often facilitate corals by maintaining low abundances of seaweeds^31^; herbivorous fish typically attain higher abundances and greater diversity of species and feeding modes in tropical reefs in comparison to temperate reefs^32–34^. Increasing abundances of herbivorous fish on temperate reefs can translate into higher rates of herbivory and a reduced abundance of seaweed through direct consumption^35^ or indirectly by affecting seaweed reproduction^36^ and recruitment^14^. This can have cascading effects that encompass multiple trophic levels, and reduce the resistance and resilience of kelp ecosystems^11, 14, 37^. Moreover, a loss or reduction in habitat-forming seaweeds can also indirectly affect other systems, such as seagrass meadows, that often receive energy and nutrients from detached seaweeds from adjacent reefs^38, 39^.

Evidence for the impact of tropical herbivorous fish in temperate marine ecosystems has been increasing in recent years^11, 40^. Tropical parrotfish, which consume seagrass at higher rates than local temperate herbivores, are increasing in abundance in the northern Gulf of Mexico^41^. In southern Japan, temperate reefs now host permanent populations of tropical rabbitfish and parrotfish^13^ which have contributed to the decline of extensive kelp forests^42–44^. Eastern Mediterranean habitats that used to be dominated by seaweeds have been transformed into barren seascapes after the intrusion and settlement of tropical rabbitfish^45, 46^ through the Suez Canal^47^. South-eastern Australia has experienced an increase of tropical surgeonfishes^48^ and rabbitfishes^35^ that have caused the decline of kelp forests on offshore reefs^35^.

In Western Australia, an exceptional marine heatwave during the summer of 2010–2011^49–51^ allowed the poleward migration and population expansion of the tropical rabbitfish *Siganus fuscescens* in the region^14, 52, 53^. This species is a characteristic herbivore of tropical reefs^54–56^, but now is an important consumer of kelp in south-eastern Australia^35^. Here we investigate if the heatwave, by allowing the expansion of rabbitfish, had indirect effects on kelp forests at latitudes lower than those at which it directly caused physiological impacts on kelp. We present a temporal analysis of changes in abundance of herbivorous fish, kelp and rates of herbivory from years prior to the marine heatwave (2004 and 2007) to the present (2016).

## Results

**Figure 1:**
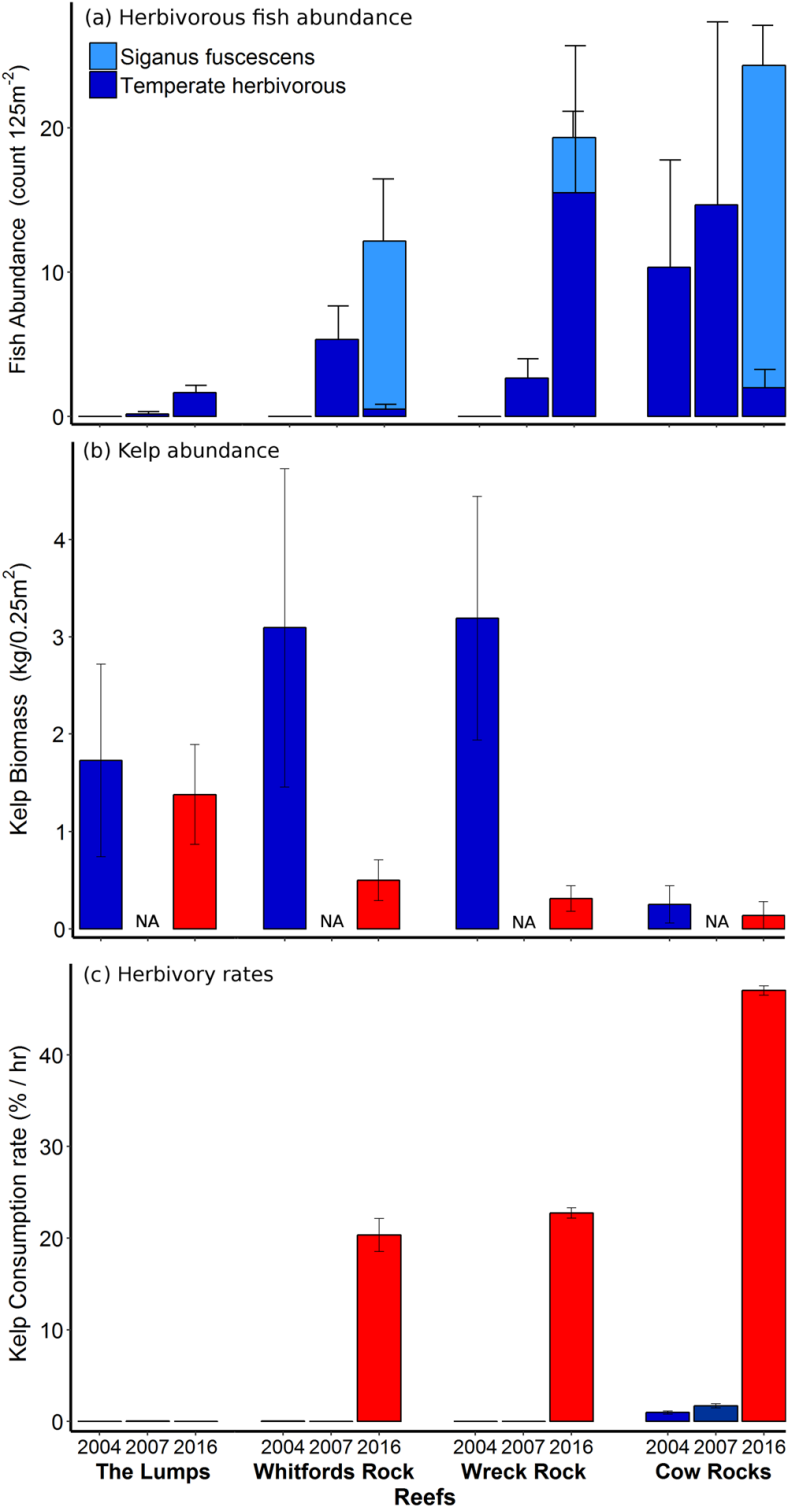
The abundance (mean ± SE) of herbivorous fish (temperate: *Kyphosus sydneyanus* and *Olisthops cyanomelas*) known to consume kelp (*Ecklonia radiata*) (**a**), kelp biomass (**b**) and herbivory rates on kelp (**c**) at temperate reefs of Marmion Marine Park, south-western Australia, from years before (2004, 2007) and after (2016) the marine heatwave of 2011.

Within the fish community, three species were identified as documented consumers of kelp: the temperate *Kyphosus sydneyanus* (silver drummer) and *Olisthops cyanomelas* (herring cale) and the tropical *Siganus fuscescens* (rabbitfish, Supplementary Table S1). Among these herbivores, *K. sydneyanus* was the most abundant species in 2004 and 2007 (2.2 ± 1.9 and 3.0 ± 2.6 individuals 125 m^−2^ [mean ± SE], respectively), followed by *O. cyanomelas* (0.3 ± 0.2 and 1.5 ± 0.7 individuals 125 m^−2^). However, abundances varied among reefs (PERMANOVA, pseudo-*F*_6, 51_ = 2.84, *P *= 0.013). Cow Rocks had the highest abundance of temperate herbivorous fish and The Lumps had the lowest, while Whitfords Rock and Wreck Rock hosted intermediate abundances. *S. fuscescens* was not recorded on any reefs in 2004 or 2007 (Fig. 1a). In contrast, *S. fuscescens* was the most abundant herbivore recorded in 2016 (9.4 ± 2.1 individuals 125 m^−2^; Fig. 1a), representing 19% of all individual fish recorded. The abundance of *K. sydneyanus* (4.7 ± 2.7 individuals 125 m^−2^) and *O. cyanomelas* (0.2 ± 0.1 individuals 125 m^−2^) did not change between years and consistently represented ~10% of the total fish abundances recorded (PERMANOVA, pseudo-*F*_2, 51_ = 1.98, *P* = 0.12). *S. fuscescens* was not recorded at The Lumps, which still had the lowest herbivore abundances; however, *S. fuscescens* was present on all other reefs, where Cow Rocks consistently had the highest abundances of herbivorous fish (Fig. 1a).

Kelp biomass decreased from 2.1 ± 0.6 kg 0.25 m^−2^ in 2004 to 0.58 ± 0.17 kg 0.25 m^−2^ in 2016, representing a loss of 1.48 kg 0.25 m^−2^ or 71% (Fig. 1b; PERMANOVA, pseudo-*F*_1, 32_ = 6.29, *P* = 0.017). Kelp declined most at Wreck Rock (84%) and Whitfords Rock (67%). At Cow Rocks, kelp abundance was low in both 2004 and 2016 (but still declined 10%), whereas at The Lumps kelp biomass remained high in both 2004 and 2016, showing no signs of decline (Fig. 1b).

Before the heatwave, there was no consumption of kelp tethers, except at Cow Rocks, where low herbivory rates were recorded (Fig. 1c). After the heatwave in 2016, kelp consumption rates were 30-fold higher than those recorded in 2004 and 2007 (Fig. 1c; PERMANOVA, pseudo-*F*_2, 78_ = 3246, *P* = 0.0001). In 2016, 5-day herbivory assays resulted in 88.8% ± 2.6 consumption day^−1^ (mean ± SE; equivalent to ca. 3.7% hr^−1^) of kelp biomass across all reefs hosting rabbitfish. Caged kelp tethers did not show any loss of biomass, supporting the inference that kelp loss was due to consumption by fish. These rates far surpassed those measured over similar time periods prior to the heatwave (20.8% ± 0.1 day^−1^ in 2004 and 20.6% ± 0.1 day^−1^ in 2007; equivalent to ca. 0.9% hr^−1^). Additional 4 hr video-filmed herbivory assays in 2016 recorded consumption rates of 28.4% ± 2.9 hr^−1^ at reefs hosting rabbitfish, suggesting most of the consumption in the 2016 five-day assays occurred at higher rates than when calculated across the entire deployment time. In contrast to The Lumps where no herbivory and no rabbitfish were recorded (Fig. 1c), Cow Rocks had the highest herbivory rates (47.1% ± 0.5 hr^−1^), followed by Wreck Rock (22.7% ± 0.5 hr^−1^) and Whitfords Rock (20.3% ± 1.8 hr^−1^) (Fig. 1c; PERMANOVA, pseudo-*F*_6, 78_ = 476, *P* = 0.001). Video-analyses of the herbivory assays confirmed that rabbitfish were responsible for all the consumption at Whitfords Rock and Wreck Rock, while consumption at Cow Rocks was shared between rabbitfish and silver drummers, which consumed approximately 50% each.

**Figure 2:**
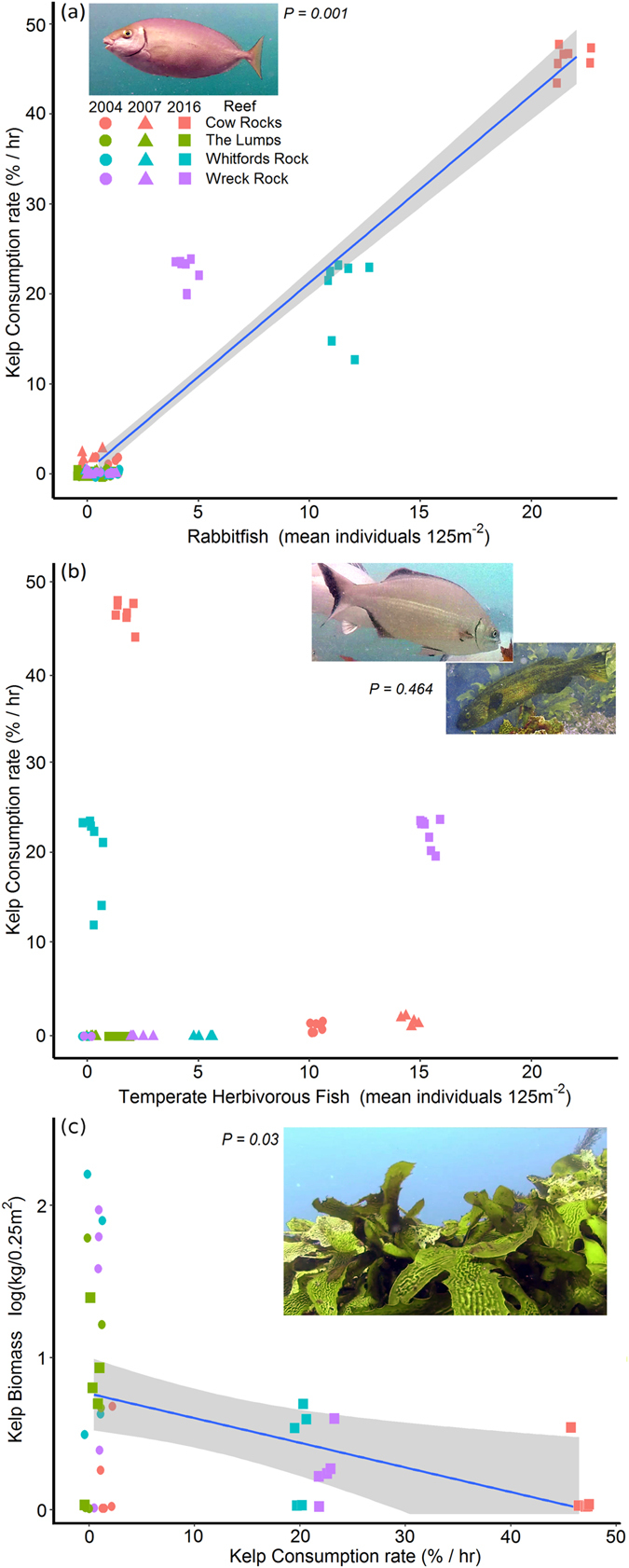
Relationship between herbivory rates and the abundance of rabbitfish (*Siganus fuscescens*), y = 1.33 + 2.05x, R^2^ = 0.89 (**a**), temperate herbivorous fish known to consume kelp (species pooled: *Kyphosus sydneyanus* and *Olisthops cyanomelas*), y = 5.88 + 0.407x, R^2^ = 0.02 (**b**), and kelp biomass (*Ecklonia radiata*), y = 1.85 − 0.0458x, R^2^ = 0.16 (**c**), on inshore reefs at Marmion, south-western Australia. Most of the points in (**a**) from before the marine heatwave (2004 and 2007) are clustered and overlapped close to zero. Regression line shown for statistically significant relationships, grey-shaded areas represent 95% confidence intervals and p-values were generated with PERMANCOVA.

There was a significant positive relationship between rabbitfish abundance and herbivory rates (Fig. 2a; PERMANCOVA, pseudo-*F*_1, 8_ = 98, *P* = 0.001); whilst there was no relationship between abundance of temperate herbivorous fish and herbivory rates (Fig. 2b; PERMANCOVA, pseudo-*F*_1, 6_ = 0.1, *P* = 0.464). In turn, there was a significant negative relationship between kelp consumption rates on tethers and standing kelp biomass on the reefs (Fig. 2c; PERMANCOVA, pseudo-*F*_1, 4_ = 11, *P* = 0.03).

## Discussion

Our results document the establishment of rabbitfish (*Siganus fuscescens*) on temperate reefs off Perth following the 2011 marine heatwave, and show a concurrent steep increase in consumption rates of kelp. The abundance and rates of herbivory by two temperate herbivorous fishes (*Kyphosus sydenayus* and *Olisthops cyanomelas*) did not change among surveyed years, indicating that the increase in the consumption rates of kelp, and decrease in kelp abundance, is likely linked to the colonizing tropical rabbitfish — an inference that was supported by the video-analyses. Rabbitfish have not displaced the temperate herbivorous fish, but rather they have been an addition to the guild. *Kyphosus sydneyanus* consumed kelp at higher rates per individual than *S. fuscescens*, but kelp consumption by rabbitfish was higher due to their higher abundances. *Siganus fuscescens* has become now the most important kelp consumer on these reefs.

The broader spatial distribution of rabbitfish in south-western Australia after the heatwave is currently unknown, but we would expect to find similar patterns of abundance and herbivory across the other extensive inshore reefs in the region. The majority of the evidence suggest that *S. fuscescens* tends to inhabit shallow near-shore reefs adjacent to seagrass meadows. Past studies have found greater abundance closer to the coast^53, 57^. Although in south-eastern Australia rabbitfish were more common on offshore reefs, their abundance was low (2.2 ± 1.8 individuals 125 m^−2^)^35^. Our results suggest that *S. fuscescens* have specific habitat preferences. Despite being <1 km away from reefs hosting abundant rabbitfish populations they were absent at The Lumps, which is a slightly deeper (~7 m vs ~5 m) more contiguous reef environment than the other reefs surveyed. This may be related to the behaviour of tropical herbivorous fish which tend to prefer habitats with lower seaweed cover^58^. Further research is required to clarify these habitat affinities to make accurate predictions of future geographic distribution changes and associated impacts with habitat-forming seaweeds.

Past studies on the tropicalization of herbivore communities have regarded rabbitfish, including *S. fuscescens*, mainly as grazers^14, 45^. However, we found that *S. fuscescens* can also be an important browser in temperate ecosystems. This is in agreement with studies from south-eastern Australia^35^ and tropical reefs (Great Barrier Reef) that found *S. fuscescens* as the main consumer of *Sargassum* ^54–56^. These differences between studies are not surprising. Although *S. fuscescens*, and herbivores in general, often feed selectively from the available food sources^59^, they usually feed on a diverse mix of primary producers^60^, allowing them to play different functional roles^57^. We did not measure grazing rates but rabbitfish were observed biting on the substrate and epiphytic algae on seagrass shoots.

This feeding multifunctionality has important implications for the potential impact that these tropical herbivores can have in temperate ecosystems. High rates of grazing by rabbitfish can reduce the recruitment of habitat-forming seaweeds by consuming seaweeds in early-life stages, as they do in tropical reefs to maintain a seaweed-free state^37, 61^. Past studies have concluded that this was the main impact of rabbitfish on temperate seaweed communities^14, 45^. However, high rates of browsing by a single species can reduce kelp biomass considerably^62^. Rabbitfish have inhabited south-western Australia for at least three years^52^ and based on our video-filmed herbivory assays they have likely been consuming kelp throughout this period. Our results suggest that the decline in the biomass of kelp on inshore reefs has occurred due to increased consumption by rabbitfish. Wherever we observed rabbitfish we found an associated increase in herbivory and a decrease in kelp biomass; where they were absent, the opposite pattern was found. In addition to the direct reduction in kelp biomass, high levels of browsing can diminish the reproductive output of seaweeds^36^; although this remains poorly understood. In the case of *E. radiata* in the region, it produces the highest number of zoospores between April and May^63^, the same time of the year when our tethered kelps were consumed at high rates. Remaining kelps on the reefs with rabbitfish showed clear signs of heavy pruning by browsers (personal observation, S. Zarco-Perello & T. Wernberg), suggesting that rabbitfish do consume, and reduce, kelp reproductive tissue, potentially causing a decline in the supply of zoospores and abundances of gametophytes that later would become kelp recruits^64^.

It is likely that other factors have acted in synergy with herbivory to cause the decline of kelp. Temperature and UV radiation have been increasing over the last decades in the region^15, 65, 66^ and both can affect survival, growth and recruitment of kelp^67–71^. The marine heatwave that impacted the region in 2011 could have caused some mortality^72^, but not at the levels observed in our results at the latitude of our study sites^8^. The slightly deeper environments at The Lumps, the only reef with consistently high abundance of kelp, could have been less affected by these physical factors, although the difference in depth is not likely to be substantial enough to attenuate high temperatures or UV radiation^67, 70^, pointing to the low herbivory rates and its historical higher levels of productivity as the cause of its higher kelp abundance^24^.

The present study provides further evidence of the consequences of the globally emerging process of tropicalization of temperate reefs^11, 40^. As the poleward-migration of marine species is becoming more accentuated due to global warming, novel biological interactions within^17, 73^ and between trophic levels are emerging^19, 74^. The alteration of species richness and abundance of consumers impact lower trophic levels, leading to changes in ecosystem functioning and structure modifications^75^. Our results show that extreme marine heatwaves not only can have immediate devastating direct effects on temperate reefs^8, 72^ but also can have longer-term indirect effects by boosting the immigration of tropical herbivores that increase herbivory rates and reduce the abundance of habitat-forming seaweeds. This contrasts with observations from other tropicalized regions of the world where the process has been more gradual, such as Japan^42, 43^, the Mediterranean Sea^45^ and south-eastern Australia^35^. With the ongoing ocean warming^65, 76^, further range expansion and population increase of *S. fuscescens* is expected. Together with other environmental disturbances, such as high temperatures, these tropical herbivores pose a growing threat to temperate reefs, increasing the likelihood of regime shifts to vegetation-free states and the associated loss of environmental services of great value to human societies from these ecosystems.

## Methods

### Location

The study was carried out at four reefs off Marmion (Perth) in south-western Australia (31°49.4 S, 115°44.0 E), where studies in 2004 and 2007 had assessed species composition and abundances of fish, rates of kelp consumption by fish, and kelp abundance^24, 38^: Cow Rocks, Wreck Rock, Whitfords Rock and The Lumps, which all have similar environmental characteristics on depth and wave exposure. These reefs are characteristic of the inshore limestone reefs that exist along the coast of temperate south-western Australia. Here, the kelp *Ecklonia radiata* and fucoids such as *Sargassum* spp. dominate the reef flora, while adjacent seagrass meadows are dominated by *Posidonia sinuosa* and *Amphibolis* spp.^24, 38^. Historically, herbivory on these reefs has been low and mainly attributed to sea urchins with fish playing a minor and localised role^24, 38^.

### Species Composition and Abundance of Herbivorous Fish

Fish surveys were undertaken in April 2004^38^ and 2007 (M. Vanderklift unpublished data), and repeated in 2016 (this study). In 2004 and 2007 surveys were done by Underwater Visual Census (UVC). In 2016 the surveys were done by Stereo-Diver Operated Video (Stereo-DOV) following standard procedures^14, 77^ in order to generate a permanent visual record. Results produced by these two techniques are comparable in temperate environments^78^. In all years and at each reef, three 25 × 5 m transects were sampled along the ecotone between reef and seagrass on each of two separate days to account for spatio-temporal variability of the fish populations. Stereo-DOV videos were analysed with EventMeasure (SeaGIS Pty Ltd) where all individual fishes were counted and identified to the lowest taxonomic level possible^14^. Fish known to be kelp consumers (Supplementary Table S1) were grouped as herbivorous and separated by climate affinity (*i.e*. tropical, subtropical, temperate) based on information published on FishBase. The abundances of these species were used in the analysis of changes in consumption rates on kelp.

### Kelp Abundance and Rates of Herbivory

Kelp abundance was quantified in 2004 and 2016 by sampling standing biomass within five quadrats (0.25 m^2^) haphazardly located at each reef, harvesting all kelp and measuring its wet weight^38^.

Rates of kelp consumption were assessed from kelp tethering assays following the methods used in previous studies at the same reefs^24, 38^. At each reef, at least seven individual ~15 cm long lateral blades of *E. radiata* were attached at the top of a 0.5 meter rod fixed to the substratum to simulate kelp canopy. In addition, three caged control tethers were deployed at each reef to test for possible non-fish sources of kelp loss, so loss could be confidently attributed to fish herbivory^24, 38^. Kelp lateral blades were photographed at the beginning and at the end of each deployment to measure the consumed area per time (% loss t^–1^) using the software ImageJ (rsb.info.nih.gov/ij/)^24^. Kelp tethers were deployed for 5 days in all years^24, 38^ and, in addition, in 2016 4-hour deployments were run at each reef while filming the consumption with video-cameras (GoPro) to refine the rates of herbivory and identify the species responsible for the consumption of kelp^14^. A HOBO pendant logger recorded a mean temperature of 21 ± 0.01 °C during the 2016 deployments.

### Statistical Analysis

We tested for differences in abundance of herbivorous fish, kelp and herbivory rates between 2016 and previous years with Permutational Multivariate Analysis of Variance (PERMANOVA^79^ using the software PRIMER 6 & PERMANOVA+ (PRIMER-E Ltd) because of its robustness against assumptions of normality and homogeneity of variances in the data. Changes in abundance of temperate herbivorous fishes known to be kelp consumers (*Kyphosus sydneyanus* and *Olisthops cyanomelas*) through time were tested with two-way PERMANOVA with planned contrasts between the years before the heatwave (2004 and 2007) and post-heatwave (2016) based on Euclidean distances calculated from square-root transformed abundance data. Changes of kelp abundance through time were tested with a two-way PERMANOVA between 2004 and 2016. Changes in herbivory rates through time were analyzed with a two-way PERMANOVA with planned contrasts as explained previously based on Euclidean distances. The effect of the abundance of herbivores on herbivory rates was analysed with a two-way PERMANCOVA (*i.e*. Permutational Multivariate Analysis of Covariance), considering rabbitfish (*S. fuscescens*) and temperate herbivorous fish abundances as explanatory covariates in independent tests, while the effect of herbivory rates on kelp biomass changes was tested with a two-way PERMANCOVA, considering herbivory rates as the explanatory covariate with Euclidean distance as the resemblance measure. All tests encompassed 9999 permutations and were performed under the same statistical design, with years and reefs as fixed factors.

## Electronic supplementary material

Documented species of temperate/subtropical and tropical herbivorous fish that consume kelp in different parts of the world.
