# Supplementary material for: Tropicalization strengthens consumer pressure on habitat-forming seaweeds
Source: Sci Rep. 2017 Apr 11;7:820. doi: 10.1038/s41598-017-00991-2 (PMC5429775; doi:10.1038/s41598-017-00991-2)
Supplement: Supplementary file 1 — Documented species of temperate/subtropical and tropical herbivorous fish that consume kelp in different parts of the world. [file 41598_2017_991_MOESM1_ESM.pdf]

# Tropicalization strengthens consumer pressure on habitat-forming seaweeds

Salvador Zarco-Perello[1], Thomas Wernberg[1], Tim Langlois[1], Mathew A. Vanderklift[2].

[1] School of Biological Sciences and UWA Oceans Institute, The University of Western Australia, Crawley (Perth) 6009, Western Australia, Australia.

[2] Commonwealth Scientific and Industrial Research Organisation (CSIRO) Oceans and Atmosphere Flagship, Indian Ocean Marine Research Centre, Crawley, Western Australia 6009, Australia.

# Supplementary Material

Table S1. Documented species of temperate/subtropical and tropical herbivorous fish that consume kelp in different parts of the world. Consumption is reported as not mentioned (n/a), grams per day (g/d), bites per hour (b $h^{-1}$), percentage of stomach content (%), percentage of length (%L) and percentage of area (%A). Analysis of herbivory was reported as anecdotal (Anec), observational (Obs), feeding rate in laboratory (Lexp) or fieldwork (Fexp) and Stomach content (StCn).

| TEMPERATE | | | | | |
|---|---|---|---|---|---|
| **Location** | **Consumer** | **Kelp** | **Consumption** | **Analysis** | **Reference** |
| USA, California | *Girella nigricans* | *Macrocystis and Pterygophora* | n/a | Anec | Harris et al. 1984 |
| | *Medialuna californiensis* | | | | |
| Spain, North | *Sarpa salpa* | *Undaria pinnatifida and Saccharina latissima* | n/a | Obs | Peteiro and Freire 2012 |
| | *Boops boops* | | | | |
| Japan, South | *Calotomus japonicus* | *Ecklonia kurome* | 131 g/d | Lexp | Kiriyama et al 2001 |
| | *Prionurus Scalprum* | | 3 g/d | | |
| | *Stephanolepis cirrhifer* | | 19 g/d | | |
| Australia, Southeast | *Olisthops cyanomelas* | *Ecklonia radiata* | 99 % | StCn | Andrew and Jones 1990 |
| Australia, South | *Syn. Caranx cyanomelas* | | 1 – 99 % | StCn | Shepherd and Baker 2008 |
| New Zealand | *Aplodactylus arctidens* | *Ecklonia radiata* | 0.4 – 2.1 % | StCn | Choat and Clements 1992 |
| | *Girella tricuspidata* | | 5.4 % | | |
| | *Girella elevata* | | 3.7 – 14.7 % | StCn | Clements and Choat 1997 |
| | *Kyphosus sydneyanus* | | 17 – 52 % | StCn | Moran and Clements 2002 |
| | *Odax cyanoallix* | | 6.8 – 84 % | StCn | Zemke- White and Clements 2004 |
| | *Odax pullus* | | 1 – 70 % | StCn | Clements and Choat 1993 |
| | | *Lessonia variegata* | 57 - 76 % | StCn | Choat and Clements 1992 |

| TROPICAL | | | | | |
|---|---|---|---|---|---|
| **Location** | **Consumer** | **Kelp** | **Consumption** | **Analy sis** | **Reference** |
| Japan | Parrotfish (no id) | *Ecklonia sp.* | n/a | Obs | Hasegawa 2010 |
| | *Calotomus japonicus* | *Ecklonia kurome* | 131 g/d | Lexp | Kiriyama et al 2001 |
| | *Thamnaconus modestus* | | 19 g/d | | |
| | *Kyphosus vaigiensis* syn. *K. lembus* | | 51 g/d | | |
| | *Kyphosus bigibbus* | *Ecklonia radiata* | < 1 % | StCn | Clements and Zemke-White 2008 |
| | *Kyphosus bigibbus* | *Eckloniopsis radicosa and Undaria pinnatatifida* | 0 – 60 % | StCn | Yatsuya et al 2015 |
| | *Siganus fuscescens* | *Ecklonia cava* | 29 – 100 %L | Fexp | Hasegawa 2010 |
| | | *Ecklonia cava, Eisenia arborea* | 0 – 100 %A | Fexp | Nimura et al. 2007 |
| | | *Ecklonia kurome* | 76 g/d | Lexp | Kiriyama et al 2001 |
| Mediterranean Sea | *Siganus luridus* *Siganus rivulatus* | *Non-kelp Macroalgae* | | StCn | Azzurro et al. 2007; Bariche et al 2009 |
| | | | | Fexp | Sala et al. 2011; Vergés, Tomas, et al. 2014 |
| Chile | *Aplocactylus punctatus* | *Lessonia trabeculata* | 13 – 49 % | StCn | Caceres et al 1994 |
| Australia, West | *Kyphosus bigibbus* | *Ecklonia radiata* | 3103 ± 1925 b $h^{-1}$ | Fexp | Bennett et al. 2015 |
| | *Kyphosus gladius* | | 173 ±101 b $h^{-1}$ | | |
| | *Siganus fuscescens* | | 98 ± 20 b $h^{-1}$ | | |
| Australia, East | *Siganus fuscescens* | *Ecklonia radiata* | >300 b $h^{-1}$ | | Verges et al. 2016 |
| | *Kyphosus bigibbus* | | >300 b $h^{-1}$ | | |
| New Zealand | *Kyphosus bigibbus* | *Ecklonia radiata* | <1 % | StCn | Clements and Zemke-White 2008 |

Table S1 References:


Andrew NL, Jones GP (1990) Patch formation by herbivorous fish in a temperate australian kelp forest. Oecologia 85: 57–68. doi:10.1007/BF00317343.

Azzurro E, Fanelli E, Mostarda E, Catra M, Andaloro M (2007) Resource partitioning among early colonizing Siganus Luridus and native herbivorous fish in the mediterranean: an integrated study based on gut-content analysis and stable isotope signatures. J Mar Biol Ass Uni King 87: 991–998. doi:10.1017/S0025315407056342.

Bariche M, Sadek R, Azzurro E (2009) Fecundity and condition of successful invaders: Siganus Rivulatus and S. Luridus (Actinopterygii: Perciformes: Siganidae) in the Eastern Mediterranean Sea. Acta Ichth Pisc 39:11-18. http://yadda.icm.edu.pl/yadda/element/bwmeta1.element.dl-catalog-9188dc81-e59d-4e7a-96db-75ff814c0dcf.

Bennett S, Wernberg T, Harvey ES, Santana-Garcon J, Saunders BJ (2015) Tropical herbivores provide resilience to a climate-mediated phase shift on temperate reefs. Ecol Let 18: 714–23. doi:10.1111/ele.12450.

Caceres CW, Fuentes LS, Ojeda FP (1994) Optimal feeding strategy of the temperate herbivorous fish Aplodactylus Punctatus: The effects of food availability on digestive and reproductive patterns. Oecologia 99: 118–23. doi:10.1007/BF00317091.

Choat, JH, Clements KD (1992) Diet in Odacid and Aplodactylid Fishes from Australia and New Zealand. Mar Fres Res 43: 1451–59.

Clements KD, Choat JH (1993) Influence of season, ontogeny and tide on the diet of the temperate marine herbivorous fish Odax Pullus (Odacidae). Mar Bio 117: 213–20. doi:10.1007/BF00345665.

Clements KD, Choat JH (1997) Comparison of herbivory in the closely-related marine fish genera Girella and Kyphosus. Mar Bio 127: 579–86. doi:10.1007/s002270050048.

Harris LG, Ebeling AG, Laur DR, Rowley RJ (1984) Community recovery after storm damage: a case of facilitation in primary succession. Science 224: 1336–38. doi:10.1126/science.224.4655.1336.

Hasegawa M (2010) Isoyake studies in Shizuoka Prefecture, Japan. Bull. Fish. Res. Agen. 32: 109–14.

Kiriyama T, Noda M, Fujii A (2001) Grazing and bite marks on Ecklonia kurome, caused by several herbivorous fishes. Suisanzoshoku 49: 431–38.

Moran D, Clements KD (2002) Diet and endogenous carbohydrases in the temperate marine herbivorous fish Kyphosus Sydneyanus. J Fish Biol 60: 1190–1203. doi:10.1111/j.1095-8649.2002.tb01714.x.

Nimura K, Takatsuji H, Masuda S, Shimamoto J (2007) Growth and maturation of Ecklonia Cava and Eisenia Arborea seedlings transplanted along the Coast of Hainan, Shizuoka Prefecture and the grazing caused by herbivorous fish Siganus Fuscescens. Aqua Science 55: 541–46. doi:10.11233/aquaculturesci1953.55.541.

Peteiro C, Freire O (2012) Observations on fish grazing of the cultured kelps Undaria Pinnatifida and Saccharina Latissima (Phaeophyceae, Laminariales) in spanish atlantic waters. AACL Bioflux 5: 189–96.

Sala E, Kizilkaya Z, Yildirim D, Ballesteros E (2011) Alien marine fishes deplete algal biomass in the Eastern Mediterranean. PLOS ONE 6: e17356. doi:10.1371/journal.pone.0017356.

Shepherd SA, Baker JL (2008) Investigator group expedition 2006: flexible foraging ecology of a temperate herbivore, the Herring Cale, Olisthops Cyanomelas, in South Australia. Trans Roy Soc S Aust 132 : 147–62. doi:10.1080/03721426.2008.10887100.

Vergés A, Tomas F, Cebrian E, Ballesteros E, Kizilkaya Z, Dendrinos P, Karamanlidis AA, Spiegel D, Sala E (2014) Tropical rabbitfish and the deforestation of a warming temperate sea. J Ecol 102: 1518–27. doi:10.1111/1365-2745.12324.

Vergés, A., Doropoulos, C., Malcolm, H. A., Skye, M., Garcia-Pizá, M., Marzinelli, E. M., ... & Bozec, Y. M. (2016). Long-term empirical evidence of ocean warming leading to tropicalization of fish communities, increased herbivory, and loss of kelp. Proc. Natl. Acad. Sci., 113: 13791-13796. doi: 10.1073/pnas.1610725113

Yatsuya K, Kiyomoto S, Yoshimura T (2015) Seasonal changes in dietary composition of the herbivorous fish Kyphosus Bigibbus in Southwestern Japan. Fish Sci 81 : 1025–33. doi:10.1007/s12562-015-0919-y.

Zemke‐ White WL, Clements KD (2004) Relationship between long‐ term changes in algal community structure and herbivore diet at the Three Kings Islands, New Zealand. New Zea J Mar Fresh Res 38: 837–44. doi:10.1080/00288330.2004.9517283.
